# DNA Damage Protection for Enhanced Bacterial Survival Under Simulated Low Earth Orbit Environmental Conditions in *Escherichia coli*

**DOI:** 10.3389/fmicb.2021.789668

**Published:** 2021-12-14

**Authors:** Jaume Puig, Nastassia Knödlseder, Jaume Quera, Manuel Algara, Marc Güell

**Affiliations:** ^1^Translational Synthetic Biology Laboratory, Experimental and Health Sciences Department, Universitat Pompeu Fabra, Barcelona, Spain; ^2^Experimental and Health Sciences Department, Universitat Pompeu Fabra, Barcelona, Spain; ^3^Radiation Oncology Department, Hospital del Mar, Parc de Salut Mar, Barcelona, Spain; ^4^IMIM Hospital del Mar Medical Research Institute, Barcelona, Spain

**Keywords:** astrobiology, DNA repair, directed molecular evolution, UV survival, radiation resistance, low pressure, outer space

## Abstract

Some organisms have shown the ability to naturally survive in extreme environments, even outer space. Some of these have natural mechanisms to resist severe DNA damage from conditions such as ionizing and non-ionizing radiation, extreme temperatures, and low pressures or vacuum. A good example can be found in *Deinococcus radiodurans*, which was exposed to severe conditions such as those listed in the Exposure Facility of the International Space Station (ISS) for up to three years. Another example are tardigrades (*Ramazzottius varieornatus*) which are some of the most resilient animals known. In this study, the survival under simulated Low earth Orbit (LEO) environmental conditions was tested in *Escherichia coli*. The radiation resistance of this bacteria was enhanced using the Dsup gene from *R. varieornatus*, and two more genes from *D. radiodurans* involved in DNA damage repair, RecA and uvrD. The enhanced survival to wide ranges of temperatures and low pressures was then tested in the new strains. This research constitutes a first step in the creation of new bacterial strains engineered to survive severe conditions and adapting existing species for their survival in remote environments, including extra-terrestrial habitats. These strains could be key for the development of environments hospitable to life and could be of use for ecological restoration and space exploration. In addition, studying the efficacy and the functioning of the DNA repair mechanisms used in this study could be beneficial for medical and life sciences engineering.

## 1. Introduction

Microorganisms and bacteria are essential for the biosphere, persevering in a wide variety of environmental conditions, some of which could be considered “extreme.” However, this definition has a strong anthropocentric criterion, since the conditions we may perceive as “extreme” are nominal and/or optimal for the growth of several species (Rothschild and Mancinelli, 2001). Furthermore, extremophilic life has constituted a large part of the evolutionary history of life, as our understanding of the environment is based on the current planetary surface conditions on Earth, which have only occurred for a short period of time compared to the existence of life (Knoll, 2015).

Some of these resilient organisms have been isolated or tested inside and outside the International Space Station (ISS), orbiting in the Low Earth Orbit (LEO), ~360 km above Earth's surface (Bijlani et al., 2021). This is the case of some species of tardigrades, such as *Ramazzottius varieornatus* (Jönsson et al., 2008), and bacteria such as *Deinococcus radiodurans* (Kawaguchi et al., 2020). In addition to these experiments, space agencies have stated that microbes are key to developing extra-terrestrial habitats. Microbes could be used for oxygen production (Leigh Mascarelli, 2010), fixing carbon dioxide (Callaway, 2019), etc., using the present conditions and atmosphere as a launch pad for an environment hospitable to life. This study focused on studying the possibilities behind the development of environments hospitable to life in conditions like those on the ISS or Mars by exploring the limits of life and the genetic mechanisms needed to expand those limits. This could not only have an impact on developing extra-terrestrial habitats and ecological restoration, but could also help study the potential of panspermia and microbe transfer, and the exploration of extinct and extant life.

The key to bacterial survival under harsh environments resides, among other aspects, in the cellular mechanisms of DNA protection and repair (Laval, 1996). With this aim, bacteria have developed several genetic and molecular mechanisms. Understanding and being able to enhance these existent mechanisms or introducing new genetic systems from resilient species is crucial not only for the development of bacteria capable to persevere under extreme conditions but could also benefit medical and life sciences engineering addressing treatments or diseases related to severe environments or conditions.

The conditions studied during this research included high doses of ionizing radiation in the form of X-rays, non-ionizing radiation in the form of UV fluence, high and low temperatures, low pressures, and vacuum. The base parameters used for this study were extracted from the *D. radiodurans* exposure at the Exposure Facility of the Japanese Experimental Module (JEM) of the ISS (Kawaguchi et al., 2016, 2020; Yamagishi et al., 2018) during the space mission “Tanpopo.” These values were compared to those measured at the equator of Mars and on Earth's surface (Table 1).

**Table 1:** Measured values for the different environmental factors studied on the Exposure Facility aboard the ISS (Kawaguchi et al., 2020), the equator of Mars (Kminek and Bada, 2006; Cortesão et al., 2021), and Earth's surface (Shahbazi-Gahrouei et al., 2013; Merino et al., 2019).

**Environmental factor**	**Exposure facility ISS *(LEO)***	**Mars *(at equator)***	**Earth's surface^a^**
UV Fluence	124−177 MJ/m^2^/year	~50 J/m^2^	-
Ionizing Radiation	232 ± 5 mGy/year	~200 mGy/year	~200 mGy/year
Temperature Range	29 ± 5~−42 ± 5°C	20~−73°C	495~−98.6°C ^b^
Pressure Range	10^−4^~10^−7^ Pa	500~1000 Pa	0.1~112 MPa

a *These parameters apply for all the planet, but are not specific to a certain region*.

b *In absence of geothermal influence, the highest surface temperature reported on Earth is ~71°C (Mildrexler et al., 2011)*.

Radiation in space mainly consists of two types: Solar Cosmic Radiation (SCR) and Galactic Cosmic Radiation (GCR) (Hellweg and Baumstark-Khan, 2007). The first one consists of low energy solar wind particles that constantly flow out of the Sun, and highly energetic solar particle events which originate from the magnetically disturbed regions of the Sun, which sporadically emit bursts of energetic charged particles (Wilson et al., 1999). This type of radiation varies depending on the distance from the emitter, in this case, the Sun. On the other hand, the Galactic Cosmic Radiation originates in Space beyond our Solar System. These ionizing radiation types can cause different kinds of DNA damage, including double and single strand breaks (DSBs and SSBs, respectively) (Kobayashi et al., 1995; Moeller et al., 2012) and base and sugar modifications (Shuryak and Brenner, 2010). Additionally, ultraviolet radiation exposure can also induce DSBs and SSBs, as well as other effects like pyrimidine dimerization (Horneck et al., 1984). In the last decades there has been a notable interest in understanding the mechanisms by which some bacteria, like *D. radiodurans*, can decrease the effects of long-term exposure to ionizing or UV radiation (Munteanu et al., 2015). Furthermore, many ionizing radiation-based treatments, common for example to treat some kinds of cancer, harm not only the targeted tissue, but also the surrounding area, limiting the effectiveness of these treatments and increasing their adverse effects. These issues highlight the urgent need for a better comprehension of the radiation protective mechanisms.

To study the effects of ionizing radiation on cellular survival, this project follows the guidelines established by Harris et al. (2009). In this article, the authors used evolved strains of *Escherichia coli* to perform a directed evolution process that led to the increase of the surviving fraction of the different strains. The dose range of the study was replicated, with levels from 0 to 3,000 Grays (Gy), much higher than the natural environmental values on Earth, which varies depending on the specific location, being in the order of ~200 milligrays per year (Shahbazi-Gahrouei et al., 2013). The 3,000 Gy exposure would equate to >12500 years of exposure at the Exposure Facility of the ISS and ~15000 years on the surface of Mars. However, different aspects must be considered. On one hand, the rate at which radiation affects the cells will have a direct impact on the effectiveness of their DNA repair mechanisms. On the other hand, both the ISS and Mars are shielded from the cosmic radiation mentioned earlier, the first one by the Earth's upper layers of the atmosphere and geomagnetic field, and the second one by its own thin atmosphere, which offers a minimal protection against cosmic radiation (Berger et al., 2020). Analyzing the survival of the cells to higher radiation levels can be beneficial to understand the DNA repair mechanisms needed for space exploration, but also to study the possibility of facing harsher environments. To expose the cells to such levels of ionizing radiation, the Radiotherapy Department of Hospital del Mar allowed the use of a *Varian True-Beam STX*^®^ accelerator, capable of generating high-energy X-rays to reach up to 3,000 Gy. The *E. coli* cells first underwent a UV fluence and an ionizing radiation exposure, after which the surviving fractions and growth rates were studied. The exposed cells were then transformed with plasmids containing genes encoding for two proteins from *D. radiodurans*: RecA and uvrD; and one from the tardigrade *R. varieornatus*: Dsup. These genes have been studied for their roles in enhancing radiation resistance and reducing DNA damage (Kim et al., 2002; Munteanu et al., 2015; Kirke et al., 2020). RecA and uvrD, from *D. radiodurans*, both have an orthologous gene in *E. coli* with a similarity of ~53.5 and ~28% at an amino acid level, respectively. RecA has been studied and demonstrated to be relevant for the *D. radiodurans*'s cellular damage repair and natural radiation resistance (Kim et al., 2002; Munteanu et al., 2015). This protein has a critical role in biological processes requiring homologous DNA pairing and recombination (Roca and Cox, 1997). UvrD is also suspected of having a big impact on this radiation resistance. However, the role of this protein to the DNA repair for *D. radiodurans*'s radiation resistance is still not clear (Munteanu et al., 2015). Dsup, from the tardigrades spp. *Ramazzottius varieornatus*, has been previously studied for its DNA repair capabilities in human and plant cells (Hashimoto et al., 2016; Kirke et al., 2020), and its effect on *E. coli*'s radiation resistance was characterized during this study. Dsup is a highly charged nuclear protein which can bind to DNA and protect chromatin from hydroxyl radicals (Chavez et al., 2019), which are commonly generated by ionizing radiation. Once the bacteria were transformed with the plasmids containing the genes encoding for the mentioned proteins, a directed evolution process was performed, studying the effect of these genes on the surviving fraction of the different strains when exposed to both ionizing and UV radiation. After this process, single colonies were selected, and their individual surviving fractions were tested once more.

The temperature of a body in Space, which is determined by the absorption and emission of energy, depends on multiple factors, including the position of the body with respect to the Sun or other orbiting bodies, size, surface, etc. (Kawaguchi et al., 2013). Consequently, the temperature range varies significantly depending on the environment the cells are in. For instance, the temperature at the Exposure Facility of the ISS varies between 29 ± 5 and −42 ± 5°C (Kawaguchi et al., 2020), but at the equator of Mars, the range can go from 20 to −73°C (Cortesão et al., 2021). Moreover, high, and low temperatures can lead to molecular damage, but also to desiccation, which can induce DSBs or SSBs (Dose et al., 1992; Yang et al., 2009), ultimately causing severe DNA damage. To induce and test the temperature resistance of the exposed *E. coli* cells, the guidelines described by Takahashi et al. (2011) were followed. In this study, they described a temperature cycles experiment designed to simulate the temperature changes outside the ISS, with 90-min cycles, which is the approximate duration of a “day” aboard the ISS, in which the temperature varies from 80 to −80°C. To analyze the cellular response to continuous exposure, multiple consecutive cycles can be performed. During this study, a similar approach was performed to test the resistance of the selected strains from the ionizing and UV radiation exposure experiments.

Low pressures and vacuum have negative effects in gene expression and cellular growth (Gasset et al., 1994). Moreover, vacuum can cause cell dehydration and desiccation, which can lead to severe damage to the DNA, but also on other cell components, such as the lipid membranes, proteins, and nucleic acids (Dose et al., 1991; Cox, 1993). Due to the wide range of DNA damage that vacuum and low pressures can cause; it has been suggested that the radiation resistance of the *Deinococcus* spp. might be partially related to or a consequence of an adaptation to prolonged desiccation (Mattimore and Battista, 1996). However, this is yet to be proven. For this study, the pressure values used during the *D. radiodurans* test before its approval for the Tanpopo mission (Kawaguchi et al., 2013, 2020) and the values estimated for Mars' equator (Cortesão et al., 2021) were considered. Ultimately, due to technical limitations, the values used in this study were a little bit higher, around 7 kPa. However, these values correspond to a ~93% vacuum and proved to have an important impact on cellular survival.

Although they are not known to cause several damages to the DNA, salinity and pH can affect the availability of water, therefore limiting the survival of microorganisms. Saline environments include a large fraction of Earth, with a range that goes from a ~3–4% salinity in marine environments, to up to 49.7% salinity in salt inclusions (Scambelluri et al., 1997), highly influencing water activities and availability, which at its turn affects microorganism proliferation and survival. The salinity range deemed optimum for isolated microorganisms ranges between 0 and 35% (Merino et al., 2019). On the other hand, the most severe pH values in which microorganisms have been isolated are at pH 0 and pH 12.5, with an optimal pH between 0.7 and 11 for most species (Merino et al., 2019). pH has a big impact on microorganisms, affecting the ability of the cells to keep a steady neutral pH to enable cellular functions and metabolism (Krulwich et al., 2011; Jin and Kirk, 2018). In this study, the survival of the selected strains was tested for several values of salinity and pH, by means of carrying out a growth analysis. This information could help understand how microorganisms can adjust to other extra-terrestrial environments with salinity or pH values different from the ones found on the ISS facilities, such as Mars or Enceladus, which can have wide ranges of both parameters (Merino et al., 2019).

## 2. Materials and Methods

### 2.1. Strains and Cell Culture

*E. coli* strain K-12 MG1655 was cultured overnight in LB medium (0.5% yeast extract, 1% peptone from casein, 1% sodium chloride) at 37°C in an incubator with shaking at 200 rpm until it reached the desired optical density (OD). When desired, cultures were supplemented with 50 μg/mL chloramphenicol. *D. radiodurans* strain R1 (ATCC 13939) was purchased from DSMZ-German Collection of Microorganisms and Cell Cultures GmbH. *D. radiodurans* was cultured overnight in medium 53 (1% peptone from casein, 0.5% yeast extract, 0.5% glucose, 0.5% sodium chloride) at 30°C in an incubator with shaking at 200 rpm until it reached the desired OD.

### 2.2. Ionizing and UV Radiation Exposures

#### 2.2.1. UV Fluence Exposure

UV Fluence exposures were performed using a *GS GENE LINKER*^TM^ *UV Chamber* (Bio-Rad). A dose of 400 mJ (~ 5 mJ/cm^2^) was used to generate mutagenesis without drastically reducing the survival rate (Shibai et al., 2017), thus increasing the efficiency of the experiment. A saturated wild-type *E. coli* culture was diluted to an Optical Density at 600 nm (OD_600nm_) of 0.0625 and grown in a 37°C shaker for ~1 h until it reached an exponential phase of OD_600nm_ 0.5 (Sezonov et al., 2007). The cell culture at exponential phase was then distributed to sterile Petri dishes (15 ml each) to amplify the exposed surface and avoid UV shielding during the UV Fluence exposure. The Petri dishes were exposed to 400 mJ of UV Fluence in triplicates. The cultures were then diluted up to 200 ml of fresh LB medium and grown overnight in a 37°C shaker until culture saturation.

#### 2.2.2. Ionizing Radiation Exposure

**Figure 1:**
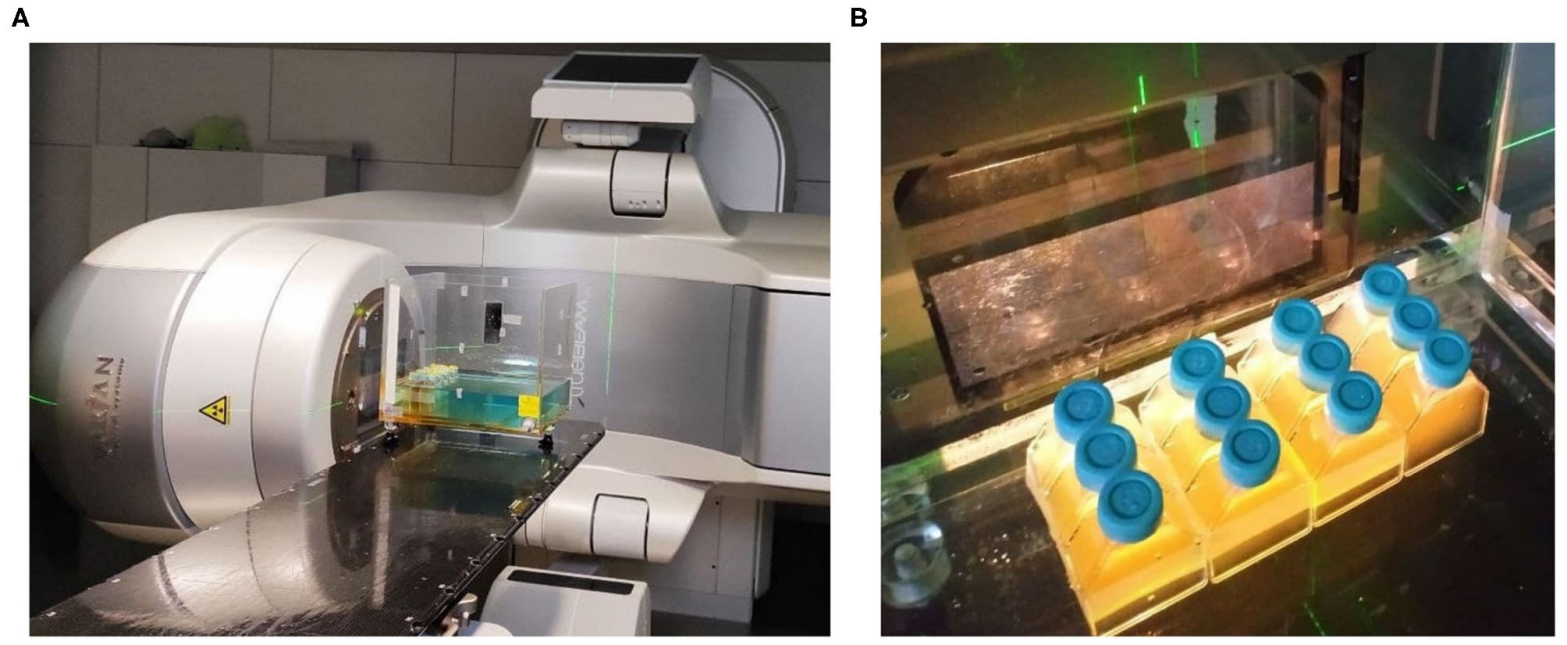
First Ionizing Radiation exposure experiment setup, featuring the *Varian True-Beam STX*^®^ accelerator used **(A)**, and the positioning of the flasks inside the water model at a Source-Skin Distance of 65 cm **(B)**.

After 18–20 h, the 200 ml saturated culture was again diluted to an OD_600nm_ of 0.0625 and incubated in a 37°C shaker for an additional hour until it reached an exponential growth phase with an OD_600nm_ of 0.5. The culture was then distributed in 13 cell culture flasks (65 ml each), one per intended IR dose. A *Varian True-Beam STX*^®^ accelerator was then used to expose the cells to a continuous spectrum of X-ray photons produced by a potential accelerator of 6 MeV (Figure 1). The studied doses were set between 0 and 3,000 Gy in steps of 250 Gy, at a dose rate of 12.6 Gy per minute and with an overall estimated error of the absorbed dose of 3.6%. During the exposure, a flask was extracted per each dose, substituting it by a flask filled with water to maintain the dose homogeneity. The survival rates were then studied following the guidelines described in the Data Acquisition Analysis section. The surviving cultures were stored at –80°C.

#### 2.2.3. Inducible Plasmid Construction and Transformation

**Figure 2:**
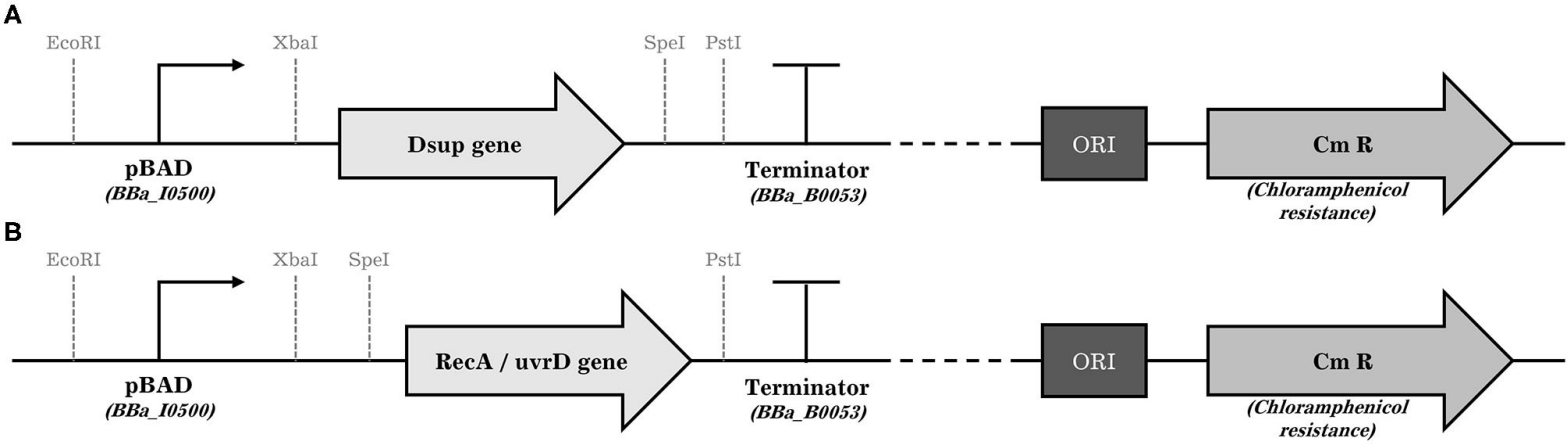
General schemes of the plasmids introduced to the surviving cells. The position of the restriction sites in the case of the Dsup gene **(A)** and the RecA and uvrD genes **(B)**, promoter, gene of interest, terminator, the origin of replication, and the chloramphenicol resistance gene is shown.

To amplify RecA and UvrR genes of *D. radiodurans*, genomic DNA was extracted by using *Bacterial Genomic DNA Isolation Kit* (Norgen Biotek Corp.) from the wild-type strain following manufacturer's instructions. Genes were amplified using *KAPA HiFi HotStart ReadyMix* (Roche) and primer pair *RecA_SpeI_fwd* and *RecA_PstI_rev*, and *uvrD_SpeI_fwd* and *uvrD_PstI_rev* (Supplementary Table S1) from *D. radiodurans* genomic DNA to add SpeI and PstI restriction sites. The Dsup gene was amplified with primer pair *Dsup_XbaI_fwd* and *Dsup_SpeI_rev* from plasmid pCXN2KS-Dsup (Addgene), (Hashimoto et al., 2016) to add XbaI and SpeI restriction sites. In summary, the PCR mix was prepared by mixing 50 ng template DNA, 0.3 μM of each primer, 12.5 μl of *KAPA HiFi HotStart ReadyMix* and H_2_O to a final volume of 25 μl. The cycling protocol was run as followed: Initial denaturation for 3 min at 95°C followed by 25 cycles of 98°C for 20 s, 61°C for 15 s, and 72°C for 2 min; and a final elongation for 2 min at 72°C. PCR products were column purified using the *QIAQuick PCR purification kit* (Qiagen). One microgram of purified DNA coding for uvrD, RecA or Dsup were subsequently digested for 1 h with either PstI, XbaI or SpeI (NEB) as required. Plasmid pSB1AC3 was digested first with EcoRI and XbaI to insert the arabinose inducible promoter pBAD/araC (iGEM Registry of Biological Parts code BBa_I0500), which was previously amplified to add EcoRI and XbaI restriction sites using the primer pair *pAra_EcorI_fwd* and *pAra_XbaI_rev* (Supplementary Table S1). Digested pSB1AC3 and digested pBAD/araC were ligated using T4 DNA Ligase (NEB). The cloning was checked using colony PCR and sequencing. In a second step, the newly acquired plasmid pSB1AC3-pBAD/araC was digested with XbaI (Dsup), SpeI (RecA, UvrD) and PstI (Dsup, RecA, UvrD) restriction enzymes depending on the gene to be inserted. Then, the previously digested genes RecA, UvrD, and Dsup were cloned downstream of the arabinose inducible promoter resulting in the final plasmids pSB1AC3-pBAD/araC- RecA, pSB1AC3-pBAD/araC- UvrD, and pSB1AC3-pBAD/araC- Dsup (Figure 2). The complete plasmids were then verified using colony PCR and sequencing.

Five milliliters of surviving cells from the first IR exposure were grown overnight in a 37°C shaker until culture saturation and grown to an OD_600nm_ of ~0.4. Competent cells were prepared by washing the cells three times with ice-cold sterile distilled water, and finally resuspended in ice-cold sterile 10% glycerol. The electrocompetent cells were transformed with the plasmids containing one of the three genes through a 2.5 kV pulse using a *Gene Pulser Xcell*^TM^ (Bio-Rad) and plated on LB plates supplemented with 50 μg/mL chloramphenicol and incubated overnight at 37°C. All colonies were picked for the following experiments minimizing the loss of population variability. The cells were inoculated in 50 ml of fresh LB medium supplemented with 50 μg/mL chloramphenicol and grown in a 37°C shaker until culture saturation.

#### 2.2.4. RNA Extraction and qPCR Analysis

Bacterial o/n cultures of *E. coli* RecA, uvrD, Dsup, and a wild-type control were diluted to a starting OD_600nm_ of 0.0625 in duplicates per sample and induced by adding 1.32 μl/ml of arabinose. After reaching an exponential phase culture, bacteria were pelleted, resuspended in 1 mL LB medium and 2 mL *RNAprotect Bacteria Reagent* (Qiagen) was added to each sample. RNA was extracted using *miRNeasy mini kit* (Qiagen) following manufacturer's instructions with some modifications. Bacterial pellet was resuspended with Trizol reagent (Qiagen), and then subjected to 45 s of bead lysis using *FastPrep FP120* at speed 6.5. Additionally, an on-column DNase digest was performed. RNA was eluted in 30 ul nuclease free H_2_0. RNA concentration was quantified using a *Nanodrop One* spectrometer and 2 μg of RNA was used from each sample to synthesize cDNA by reverse transcription using a *High-Capacity cDNA Reverse Transcription Kit* (Applied Biosystems) following manufacturer's instructions. The obtained cDNA was diluted 20 times whereof 4 μl were used per reaction. Each reaction was supplemented with 6 ul of Mix containing 5 μl *PowerUp SYBR green MasterMix* (Applied Biosystems) complemented with 0.5 μl (500 nM) of each primer fwd and rev. qPCR was performed in three technical replicates using dedicated primers against each gene and an endogenous control targeting the *E. coli* RecA gene. All primers can be found in the Supplementary Table S2.

#### 2.2.5. Directed Evolution Process

The saturated 50 ml cultures from the *E. coli* strains wild-type K-12, an exposed control, RecA, uvrD, and Dsup producing strains were exposed to Ionizing Radiation up to 3,000 Gy by following the same guidelines as in the previous exposure, diluting the cultures up to 300 ml each and incubating them until they reached an exponential growth phase. When diluted, the cultures were induced with 1.32 μl/ml of arabinose to activate the expression of the genes encoding RecA, UvrD and Dsup (Gonzalez-Flo et al., 2020). However, due to space restrictions and to maintain dose homogeneity, the studied doses were reduced to 0, 500, 1,500 and 3,000 Gy, at a dose rate of 12.6 Gy per minute. Besides, due to technical constraints, between the 500 and the 1,500 doses, a pause of 20–30 min was done. The cells were exposed up to two times, studying the impact and efficacy of the gene products, as well as the response of the cell strains to a continuous exposure to high levels of Ionizing Radiation. During the second exposure, a wild-type *D. radiodurans* culture in an exponential growth phase was also exposed. In each exposure, the surviving fraction was obtained following the guidelines described in the Data Acquisition Analysis section.

#### 2.2.6. Colony Selection

Following the last Ionizing Radiation exposure, the growth of a total of 138 colonies was analyzed, both with and without a previous exposure to 400 mJ of UV Fluence. This exposure was performed following the guidelines established in the previous UV exposure, without inducing the genes. The analyzed colonies were 12 from the wild-type *E. coli* strain, 6 from the UV-exposed *E. coli*, 12 from the control strain, 26 from the RecA strain, 30 from the uvrD strain and 52 from the Dsup strain. The growth rate analysis was studied using an *Infinite M NANO+* Plate-Reader (TECAN) to measure the OD at 600 nm, starting with an OD_600nm_ < 0.1. From the data obtained, the best performing colonies of the RecA (1), uvrD (1), and Dsup (3) strains were selected.

#### 2.2.7. Final Ionizing and UV Radiation Survival Tests

The three selected colonies of the Dsup strain, which had the biggest survival rates in the previous radiation exposures, were exposed a fourth time to study the final Ionizing Radiation resistance of these strains. The exposure was done following the guidelines of the previous exposures, but only exposing to 0 and 500 Gy. The exposed samples included the three colonies, strains C15, C16, and C17, and the surviving population from the last IR exposure.

To test the UV Fluence survival of the new *E. coli* strains, a culture of each strain including the selected colonies, the populations and the wild-types *E. coli* K-12 and *D. radiodurans* were inoculated and grown overnight at 37 or 30°C, respectively. The cultures were then diluted to an OD_600nm_ of 0.0625, induced with 1.32 μl/ml of arabinose, and grown until an exponential growth phase. The cultures were transferred to sterile Petri dishes and exposed to different doses of UV Fluence using a *GS GENE LINKER*^TM^ *UV Chamber* (Bio-Rad), exposing the cells to up to 785 mJ (~ 10 mJ/cm^2^), which corresponds to a ~ 30% of the UV Fluence on the ISS Exposure Facility per minute. This value was selected according to the technical limitations and was considered when analyzing the survival results. The surviving fraction was then obtained following the guidelines described in the Data Acquisition Analysis section.

#### 2.2.8. Whole Genome Sequencing and Analysis

From the selected colonies for the RecA, uvrD and Dsup strains, a gDNA extraction was performed using the *Bacterial Genomic DNA Isolation Kit* (Norgen Biotek Corp.). Whole Genome Sequencing (WGS) of the colonies was then conducted by the *Microbial Genome Sequencing Center* (Pittsburgh, PA) and the sequences were compared with the wild-type genome of *E. coli* K-12 MG1655. The analysis was done using the *breseq* software from Barrick Lab (The University of Texas at Austin, 2014) (Deatherage and Barrick, 2014).

### 2.3. Low Pressure and Temperature Exposure

#### 2.3.1. Low Pressure and Vacuum Simulation

The low-pressure conditions were achieved by using a desiccator coupled to a vacuum pump applying a negative pressure of –700 mmHg, exposing the cells to a pressure around 7 kPa. The cells were grown overnight in a 37 or 30°C shaker according to their optimal growth temperature. The RecA, uvrD, and Dsup strains were grown with and without inducing the genes. After 18–20 h of growth, the OD_600nm_ was measured to be around 1, and the cultures were distributed in 1 ml tubes. Two tubes were prepared by strain, one was left as a liquid culture, and the other was centrifuged at 10,000 × g and pelleted, removing the supernatant and drying the pellet. After the sample preparation, the tubes spent 0, 3 or 7 days in the desiccator at a pressure ~7 kPa, after which they were prepared for the temperature cycles experiment.

#### 2.3.2. Temperature Cycles Simulation

Following the guidelines established by Takahashi et al. (2011), the temperature cycles experiment was designed to reach from 50 to –80°C in 90 min cycles, simulating each temperature using a thermal bath and a freezer respectively. Each cycle consisted of cooling the cells at –80°C for 20 min, leaving them at room temperature for 25 min, heating up to 50°C for 20 min and finally leaving them at room temperature for an additional 25 min. For each low-pressure sample, 0, 1, and 3 temperature cycles were performed, performing up to 5 temperature cycles in the case of the 7 days low pressure exposure. After the completion of their respective low pressure and temperature cycles exposures, the surviving fraction was obtained following the guidelines described in the Data Acquisition Analysis section.

**Figure 3:**
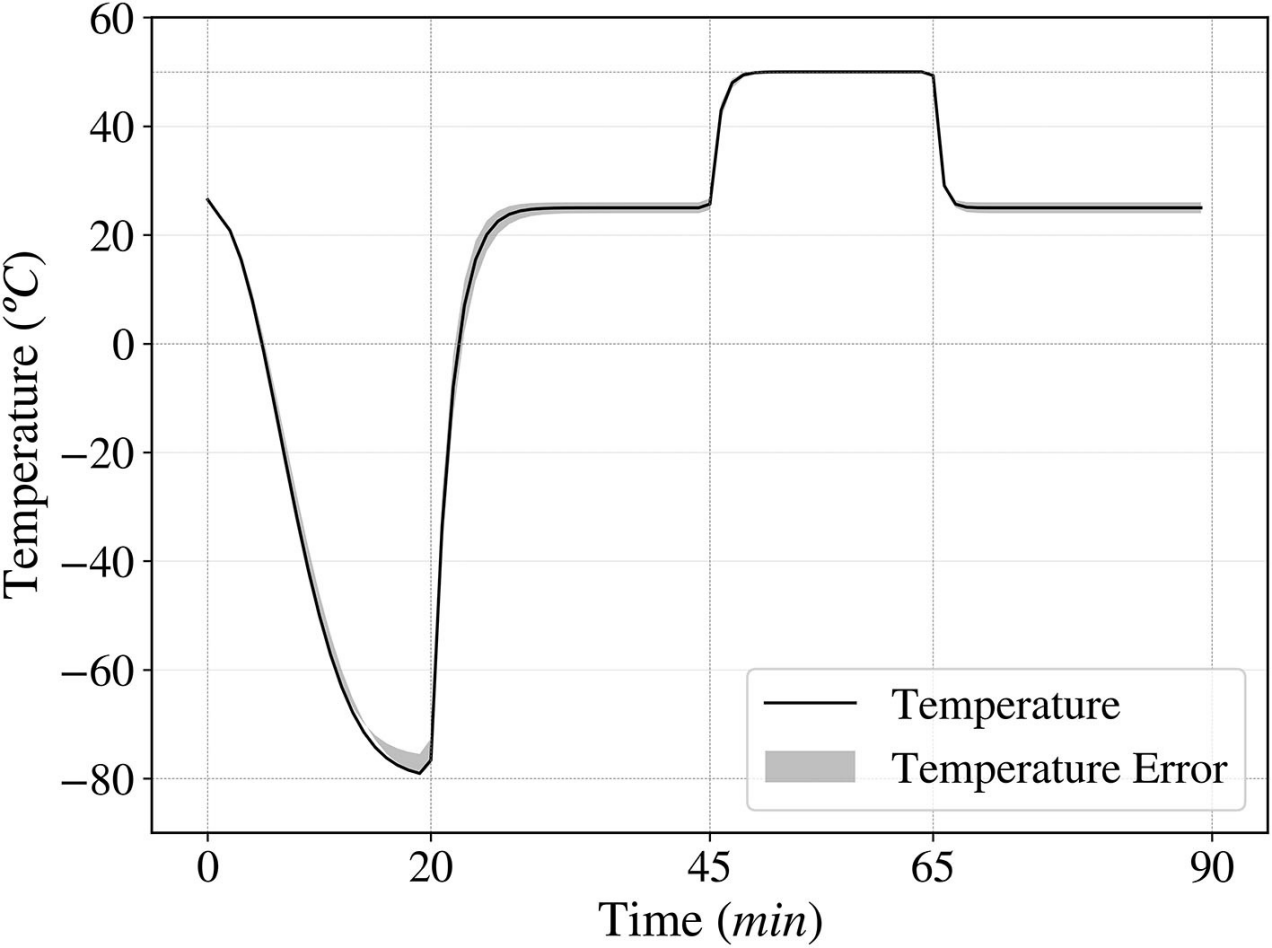
Temperature fluctuation over time in one cycle, ranging from –80 to 50°C. A complete cycle consists of 20 min at –80°C, 25 min at room temperature, 20 min at 50°C and 25 min at room temperature.

To study the temperature variation of the samples during each cycle, Newton's law of cooling was used, calculating the proportionality constant *k* experimentally. The resulting plot in Figure 3 shows how the temperature varied over time during each cycle.

### 2.4. Salinity and pH Cultures

The growth of the selected colonies from the RecA, uvrD, and Dsup strains, as well as wild-type *E. coli* were tested when different salinity and pH conditions were present on the media. An *Infinite M NANO+* Plate-Reader (TECAN) was used to measure the OD_600nm_ of the liquid cultures during >20 h, studying their growth rates. The cultures were grown overnight in a 37 or 30°C shaker according to their optimal growth temperature and were then used to inoculate 200 μl of fresh LB media with the different pH or salinity conditions in a 96-well plate, which was then used for the OD_600nm_ measurements. The Plate-Reader was set at 37°C with shaking, and the OD_600nm_ was measured every 10 min for >20 h.

The LB media was prepared with different concentrations of salt, ranging from 0 to a 57% of NaCl with respect to the rest of the media ingredients, or pH, with values between 3 and 11, using HCl and NaOH to compensate the acidity and alkalinity until the desired pH values were reached.

### 2.5. Data Acquisition and Analysis

The surviving fraction of the exposed cultures for the different conditions studied was calculated by the Colony-Forming Units (CFU) of each exposed culture. The cultures were first serially diluted up to 10^-6^ and nine drops of 5 μl per each dilution were placed in three separate LB agar plates to have triplicates. The surviving fraction of each strain was calculated from the titer of the surviving population, which was calculated from the CFU of the culture one day after the exposure, divided by the titer of the non-exposed culture, the control dose. The errors were also calculated from this data, dividing the standard deviation by the square root of the number of drops counted per sample.

In the Ionizing Radiation exposures, the linear-quadratic model was used to study the surviving fractions (Brenner et al., 1998). The model, seen in Equation (1), was fitted to the experimental data using Python programming language and the Lethal Doses for the 90% of the population (LD10) were calculated.


(1) $$\begin{array}{r} S\left(D\right)=e^{-\alpha D-\beta D^{2}} \end{array}$$

## 3. Results

### 3.1. Enhancing Ionizing Radiation Resistance

**Figure 4:**
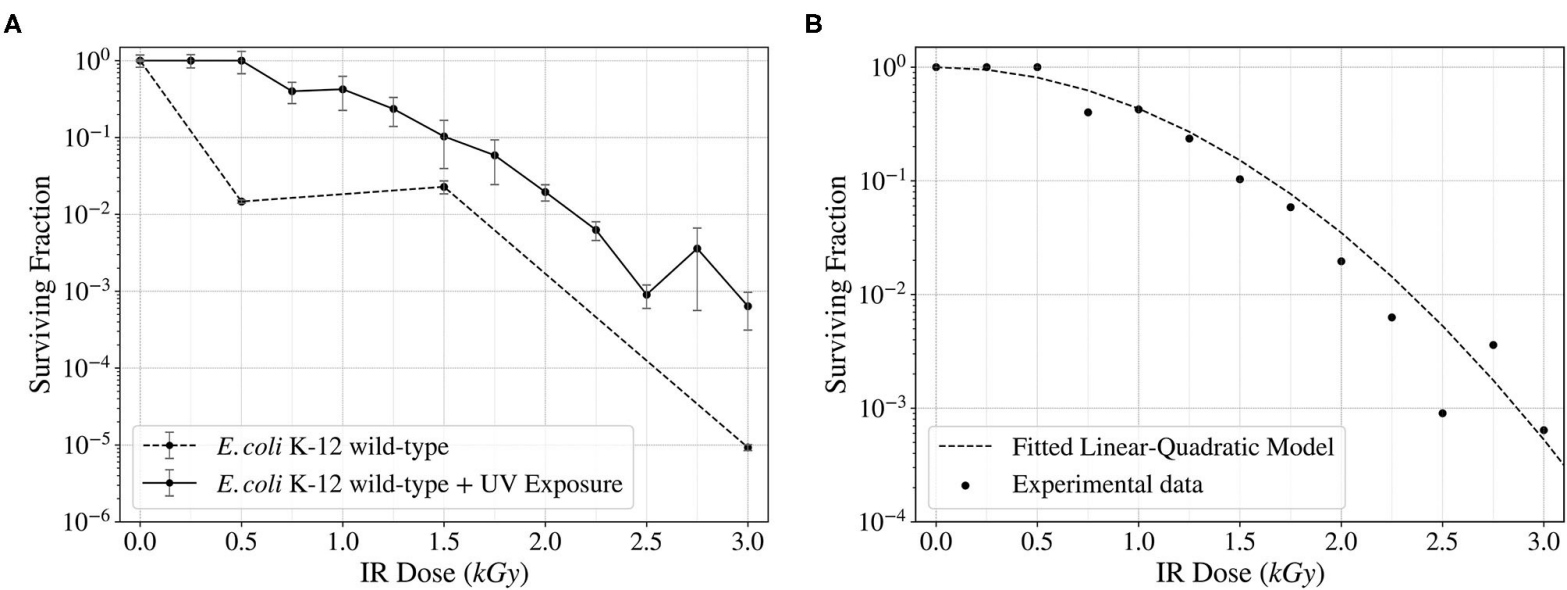
Surviving fraction of wild-type *E. coli* K-12 cells after being exposed to a continuous spectrum of X-rays (Ionizing Radiation) up to 3,000 Gy (12.6 Gy/min). Comparison of the surviving fraction with and without a previous UV Fluence exposure **(A)** and fitted linear-quadratic model showing the trend of the surviving frequency **(B)**.

Ionizing Radiation can cause different kinds of DNA damage, including DSBs and SSBs (Kobayashi et al., 1995; Moeller et al., 2012), base and sugar modifications (Shuryak and Brenner, 2010), etc. In addition, non-ionizing radiation such as ultraviolet can also induce such effects, like pyrimidine dimerization (Horneck et al., 1984). Protecting the DNA against this damage and repairing it is the key to enhancing the resistance to radiation exposures. To determine the survival of wild-type *Escherichia coli* to ionizing radiation, random mutagenesis was induced, and the cells were exposed to X-rays. These exposures showed an increase in survival in more than one and a half orders of magnitude when previously exposed to UV fluence compared to the wild-type control (Figure 4). The data obtained was fitted to a linear-quadratic model, used to simulate cellular survival when exposed to radiation (Brenner et al., 1998). The fitted data showed a good correlation between the experimental points and the mathematical model (Figure 4).

**Figure 5:**
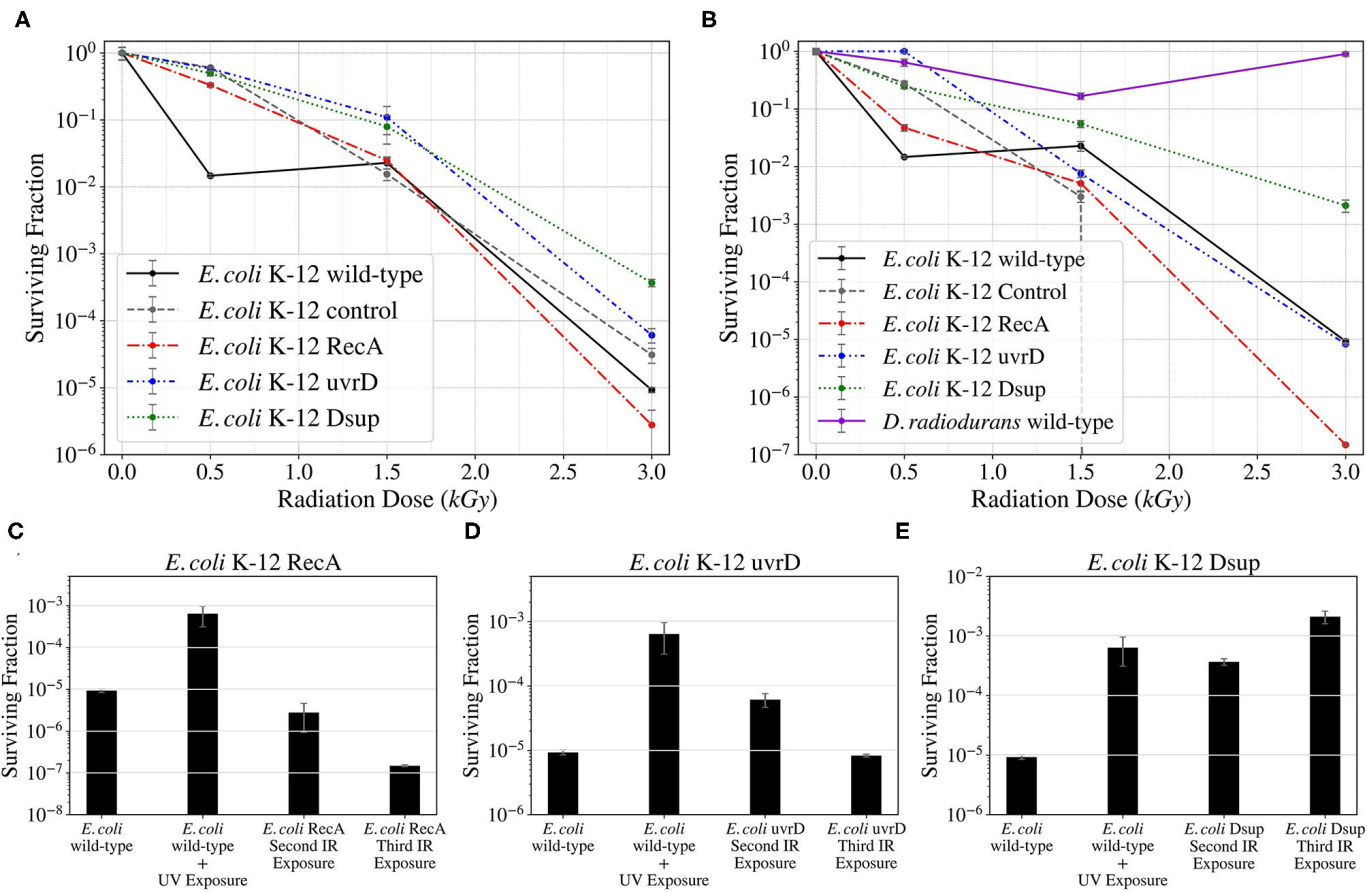
Survival curves for the different *E. coli* strains and *D. radiodurans* for the second **(A)** and third **(B)** Ionizing Radiation exposures as part of the directed evolution process; and progress bars of the survival of the RecA **(C)**, uvrD **(D)**, and Dsup **(E)** strains through the different Ionizing Radiation exposures.

After being transformed with the genes encoding for RecA, uvrD, and Dsup, the cells were then exposed twice to Ionizing Radiation, studying the surviving fraction after each exposure and analyzing the progress obtained with the different genes. In Figure 5, the surviving fractions of the different strains for the second (A) and third (B) X-ray exposure are shown. While the control and the RecA strain decreased their survival, uvrD had a similar survival as the wild-type. The Dsup strain increased the survival in more than 2 orders of magnitude with respect to the wild-type in the 3,000 Gy dose, increasing progressively throughout the exposures. *D. radiodurans* obtained a very high survival rate at 3,000 Gy.

This data was fitted using the linear-quadratic model, and the Lethal Dose that would kill 90% of the population (LD10) was calculated for the three evolved strains and the wild-type control. The Dsup strain had a LD10 around 1.68 kGy, while the wild-type and the uvrD strains were both around 1.09 kGy. The RecA strain obtained the worst results, with a LD10 of ~ 0.98 kGy.

**Figure 6:**
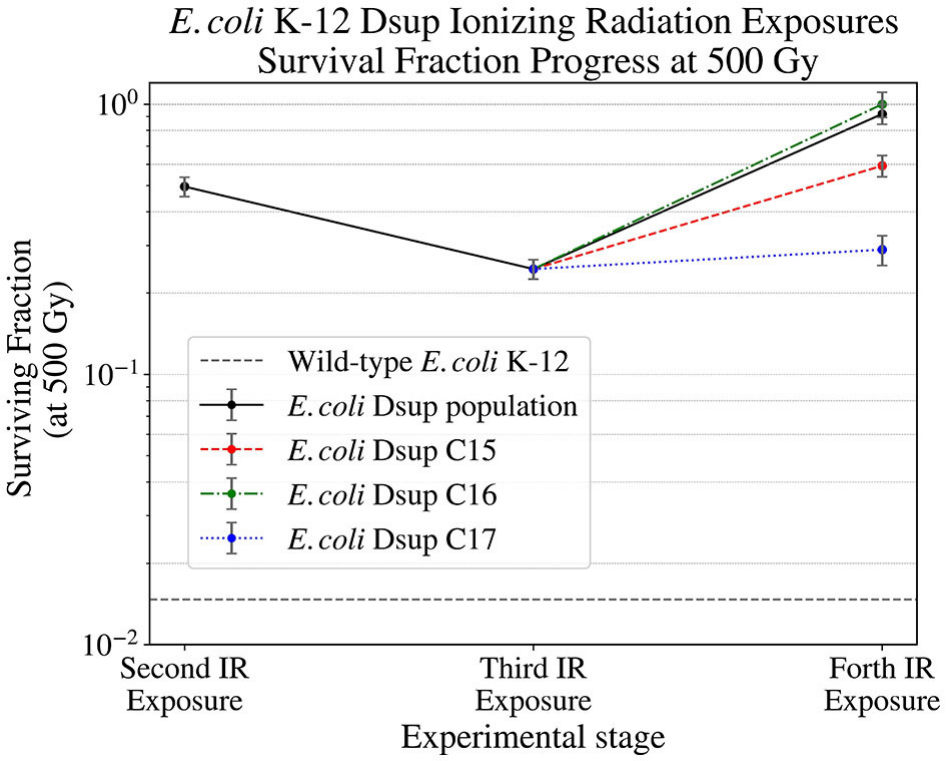
Surviving fraction of the *E. coli* Dsup strain at 500 Gy (12.6 Gy/min) during the second, third, and final Ionizing Radiation exposures, showing the progress on the survival of this strain and its selected colonies.

After selecting single colonies from the RecA, uvrD, and Dsup strains, the colonies selected from the Dsup strain were exposed to Ionizing Radiation up to 500 Gy, analyzing the survival of the colonies and the population. After this exposure (Figure 6), some colonies showed better results, but all significantly increased the survival with respect to the wild-type *E. coli*. Dsup C16 strain had the best performance, with almost a total cellular survival after the 500 Gy exposure.

**Figure 7:**
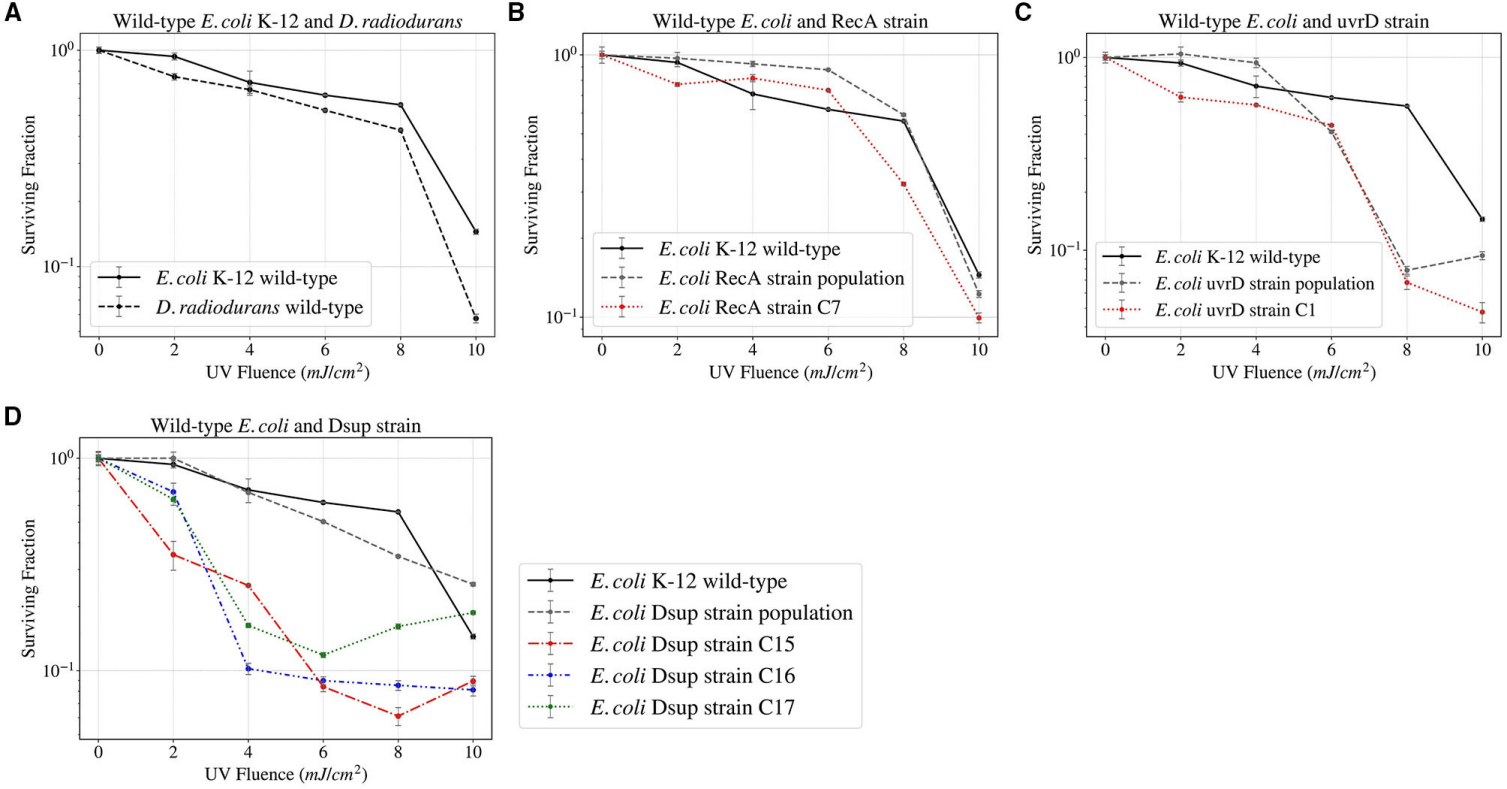
Survival curves of the different *E. coli* and *D. radiodurans* strains after UV Fluence exposure up to 785 mJ.

The *E. coli* and *D. radiodurans* wild-types, together with the RecA, uvrD, and Dsup strain populations and single colonies were finally exposed to UV Fluence up to 785 mJ (~ 10 mJ/cm^2^, Figure 7). The results showed a similar survival between the *E. coli* and *D. radiodurans* wild-types. The rest of the *E. coli* strains also showed similar survivals, with no relevant increase or decrease in any case.

The Whole Genome Sequencing (WGS) and analysis performed for a total of 5 colonies from the *E. coli* strains RecA (1), uvrD (1), and Dsup (3), which can be seen in the Supplementary Table S2, revealed more differences in the DNA repair efficiency of the three genes, with Dsup showing a very high efficacy and the C15 strain having no relevant genetic mutations with respect to the wild-type control. uvrD showed the lowest impact on preventing DNA damage, with a higher number of mutations than the other strains. From the sequencing results obtained, no relevant mutations can be seen that could unequivocally explain an increase in survival under high levels of radiation.

### 3.2. Vacuum and Temperature Testing

**Figure 8:**
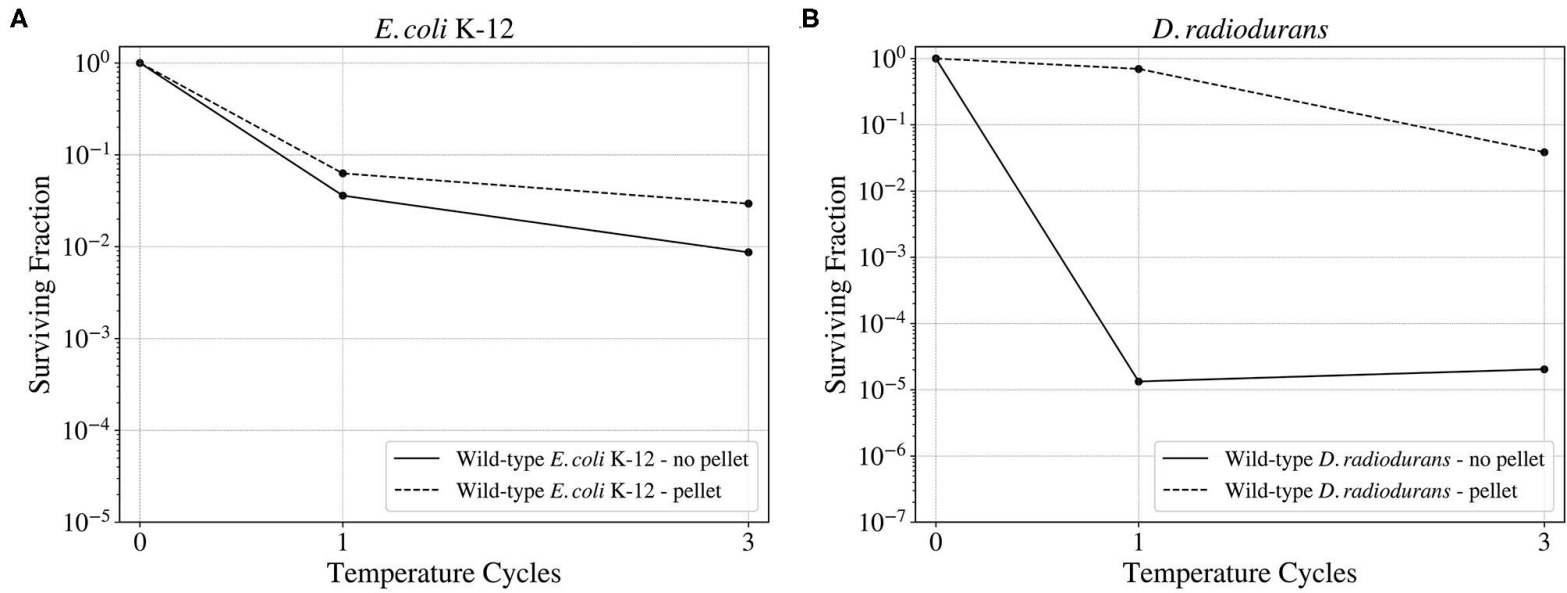
Surviving fraction of the wild-type *E. coli* K-12 cells **(A)** and *D. radiodurans* **(B)** after a 3-day exposure to a ~93% vacuum and 3 temperature cycles from –80 to 50°C. Comparison between the survival of the aggregated and the non-aggregated cultures.

Vacuum and high and low temperatures can lead to desiccation, causing severe damage to the DNA, such as DSBs and SSBs (Dose et al., 1992; Yang et al., 2009), but also to other cell components (Dose et al., 1991). Before exposing the cells to a series of temperature cycles from –80 to 50°C in 90 min, the cells were either exposed to 0, 3, or 7 days to low pressures in the order of 7 kPa. In this study, the effect of cellular aggregation to enhance survival was tested, exposing the cultures both pelleted and non-pelleted. As shown in Figure 8, in the case of *D. radiodurans* (B), cell aggregation had a big impact on survival, with an increase of the surviving fraction when the culture was pelleted. However, in the case of *E. coli* (A), cell aggregation did not have a significant impact on the surviving fraction, with a little increase of survival in the case of the pelleted culture.

**Figure 9:**
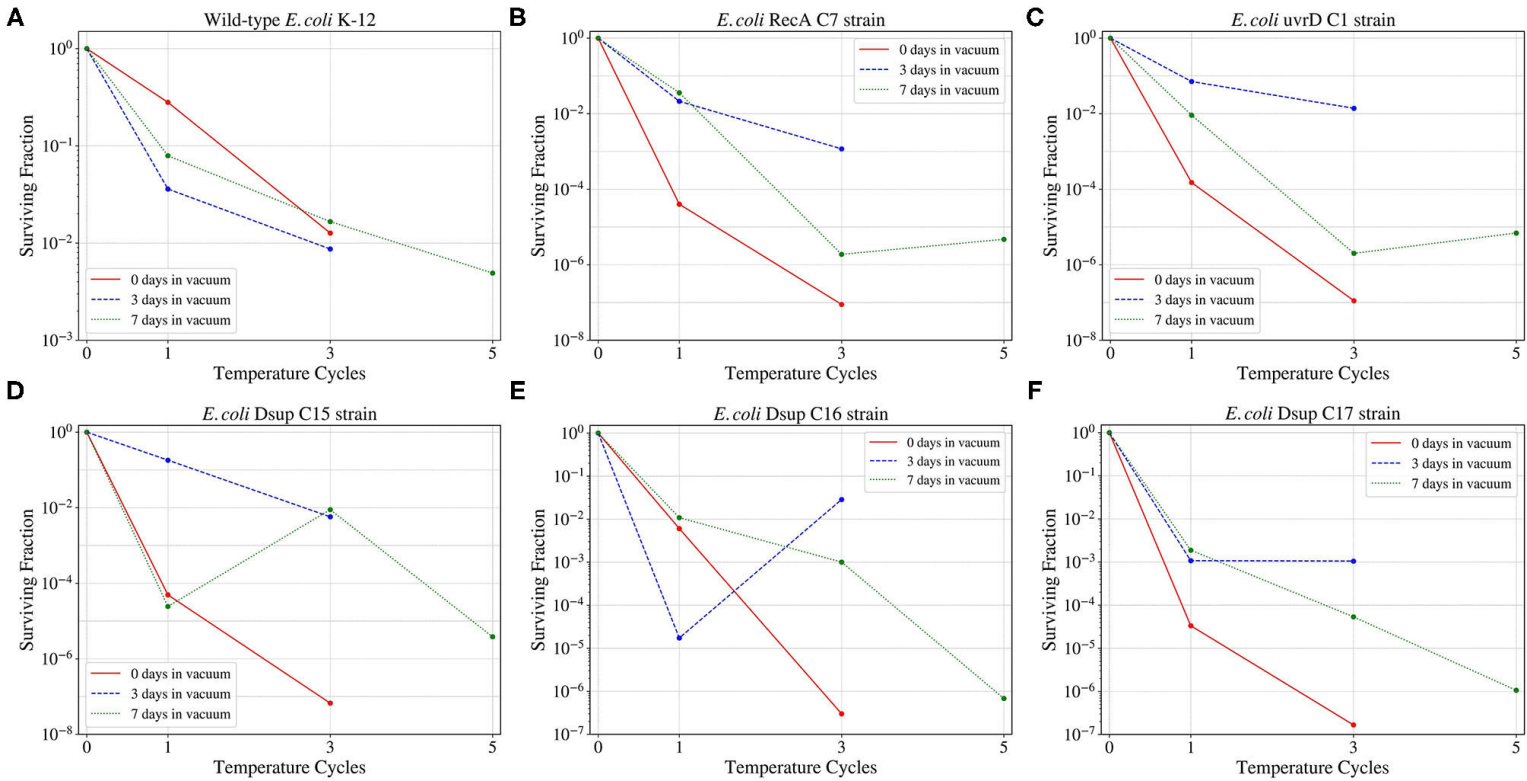
Survival of the wild-type *E. coli* K-12 strain **(A)** and the different RecA **(B)**, uvrD **(C)**, and Dsup **(D–F)** selected colonies to the temperature cycles from –80 to 50°C after 0, 3, 7 days exposures to a ~93% vacuum.

When studying the survival of the wild-type *E. coli* and the selected colonies from the RecA, uvrD, and Dsup strains to the different low-pressure exposures and temperature cycles (Figure 9); the wild-type had the highest survival in the three cases. However, for the RecA, uvrD, and Dsup strains the overall survival depended on the low-pressure exposure times, with better survival after the 3-day exposure than the 0 or the 7 days. The three strains had similar surviving fractions, with no relevant improvement in cellular survival for the tested conditions.

### 3.3. Salinity and pH Testing

Salinity and pH can have a big impact on the growth and survival of cells, mainly affecting the availability of water, and therefore cellular metabolism. The strains obtained during the previous experiments were tested in different salt concentrations and pH values to study their growth in these conditions.

**Figure 10:**
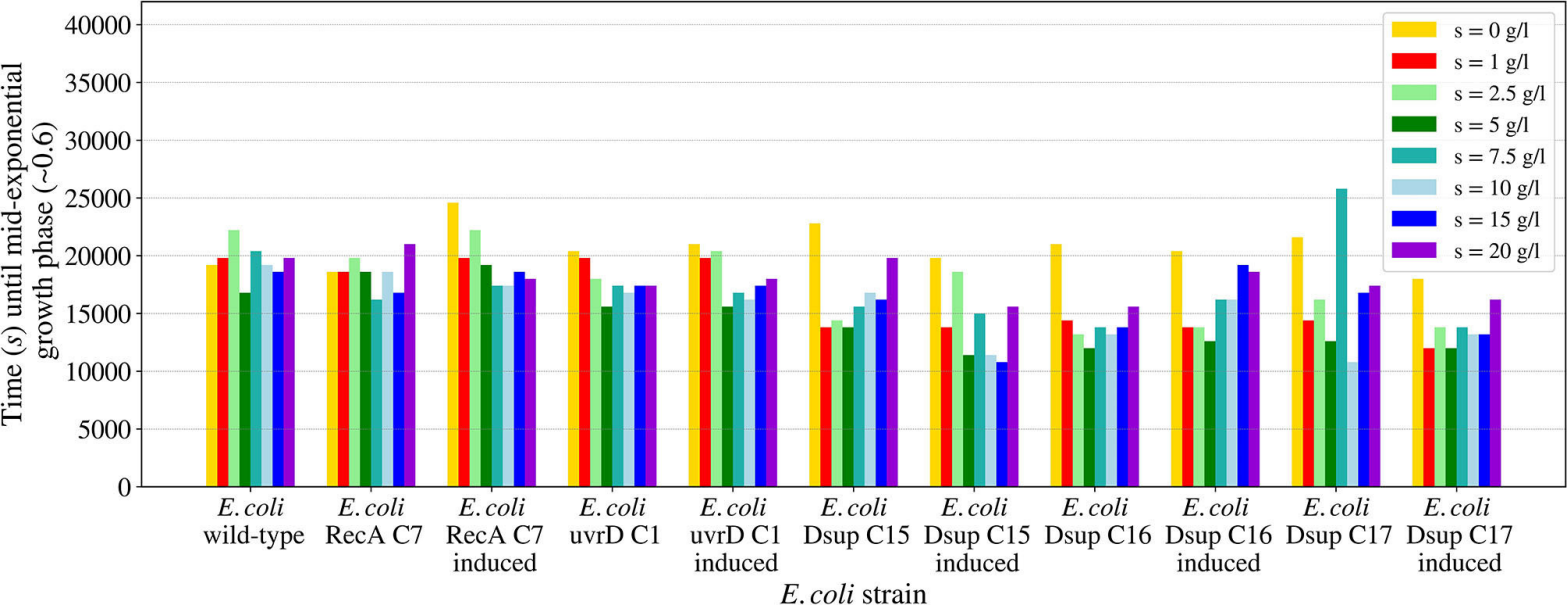
Time needed for the *E. coli* wild-type and the selected colonies of the RecA, uvrD, and Dsup strains to reach mid-exponential growth phase (OD_600nm_ ~0.6) when inoculated in LB media with different salinities. The effects of the gene products were tested with and without induction to detect any possible effects on cellular growth.

The salinity growth rate analysis (Figure 10) revealed that all the different strains tested could persevere and survive for all the conditions, therefore the known limits for these strains would be from 0 to 20 g per liter of LB media. The results were very similar between the different strains, with a faster growth for salinity around 5 g/l and slight variations for the rest of concentrations. In general, the Dsup strains showed a faster growth in most of the salinity conditions, with a small difference with respect to the rest of the tested strains.

**Figure 11:**
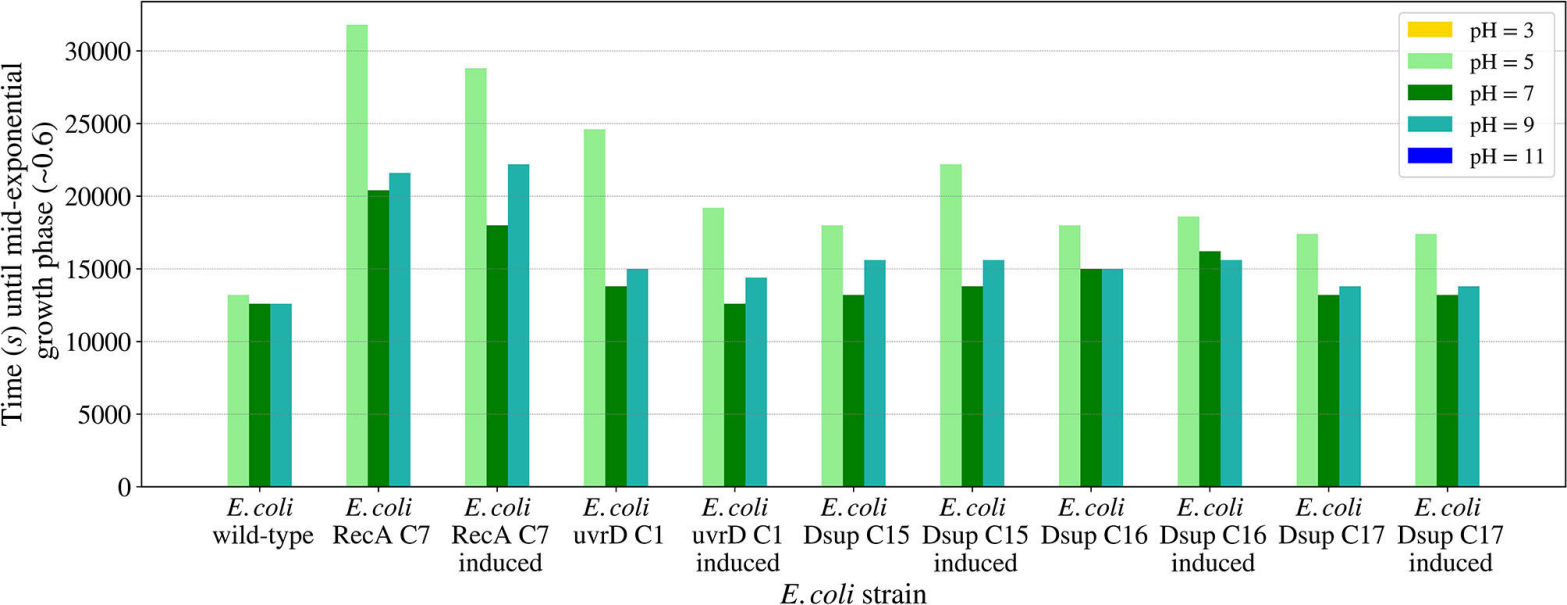
Time needed for the *E. coli* wild-type and the selected colonies of the RecA, uvrD, and Dsup strains to reach mid-exponential growth phase (OD_600nm_ ~0.6) when inoculated in LB media with different pH values. The effects of the gene products were tested with and without induction to detect any possible effects on cellular growth.

The pH growth analysis (Figure 11) revealed the importance of a neutral pH for cellular growth, with no growth for the pH values 3 or 11 for any of the tested strains. For pH values between 5 and 9, all the strains were able to grow, with a slower growth in the case of the RecA strain. The rest of the strains showed similar results, with a faster growth at pH 7. In all the cases, growth with pH 5 was slower than pH 9, showing a bigger effect of acid pH values to cellular growth.

## 4. Discussion

The initial ionizing and non-ionizing radiation exposures showed an increased survival when a first exposure to UV fluence was performed compared to a lack of the non-ionizing radiation exposure. This increase, which was of about 1.5 orders of magnitude with respect to the non-irradiated wild-type control, may be attributed to the mutagenesis and phenotypic adaptation generated by this exposure, but also to the filtering of the most vulnerable cells. The survival curve was fitted using a linear-quadratic model, and the experimental data followed the model as expected. Despite that, this increase was not maintained after selecting the surviving fraction, as can be seen in the control samples in Figure 5.

The different *E. coli* strains transformed with a plasmid containing one of the *D. radiodurans* or *R. varieornatus* genes revealed a big difference in the efficacies of the gene products in *E. coli*, as well as a big variation in the surviving fraction. This variability can be seen in Figure 5. The strain containing the RecA gene, from *D. radiodurans*, showed a progressive decrease in the surviving fraction. The strain containing the uvrD gene, from *D. radiodurans*, showed similar survival values to those obtained with the wild-type *E. coli* K-12. These lack or detrimental effects could be due to the interference with the orthologous gene of *E. coli*, with which they have a 53.5 and a 28% of similarity in an aminoacid level respectively. RecA is a central enzyme of DNA repair, and has been transplanted from pseudomonas to *E. coli* affecting DNA repair capacity (Baitin et al., 2006). However, the case of *D. radiodurans*, a more phylogenetically distant species, may be more complex. Indeed, it has been shown recently that in order to transfer effectively recombinogenic properties further modifications are required. For instance, compatible pairs of recombinases (phage origin in this case) and single stranded binding proteins (SSB) were required (Filsinger et al., 2021). RecA has a very close and dynamic relationship with SSB, displacement of the latter is modulated by RecA C-terminal (Eggler et al., 2003). *D. radiodurans* has a very unusual SSB which is twice the size and acts as a dimer instead of the classical tetramer (Ngo et al., 2013). Future plans of this project will include co-transformation with *D. radiodurans* RecA together with SSB. Unlike in *E. coli*, uvrD has a key role in *D. radiodurans*. Again, uvrD activity is strongly modulated by SSB (Stelter et al., 2013). Future work, will also include co-transformation with this protein. The Dsup gene, from *R. varieornatus*, showed an increase in more than two orders of magnitude in the surviving fraction, effectively enhancing *E. coli*'s resistance to radiation exposure after 3 X-rays exposures. The experimental data was fitted to a linear-quadratic model, allowing the full characterization of the survival throughout the dose span tested, but also the calculation of the Lethal Doses, after which 90% of the cells died (LD10). The LD10 of the wild-type *E. coli* was ~ 1.09 kGy, while for the Dsup strain, the LD10 after the three X-rays exposures was ~ 1.68 kGy, increasing in almost 600 Gy. In the case of the RecA strain, the LD10 was ~ 0.98 kGy, lower than the one for the wild-type. The uvrD strain had a LD10 similar to the one for the wild-type *E. coli*. These values confirm the significant enhancement of the survival for the Dsup strain with respect to the wild-type, proving the capacity of the Dsup protein to protect the DNA from the hydroxyl radicals that can be caused by the exposure to X-rays and the corresponding effect on cellular survival.

A final test of the surviving fraction of three selected colonies and the population of the Dsup strain when exposed to X-rays up to 500 Gy showed an almost total survival for this dose in one of the colonies and a generalized increase in the survival throughout the different ionizing radiation exposures (Figure 6). The 500 Gy exposure would equate to ~ 2500 years of exposure on the Exposure Facility of the ISS. Furthermore, increasing the time span until the same dosage is reached could have a direct positive impact on cell survival, allowing the DNA repair mechanisms to act with higher effectiveness. The Whole Genome Sequencing (WGS) results showed a higher efficiency of the Dsup gene in DNA protection, with similar results for the RecA colony, whereas the uvrD gene showed the lowest efficiency. The UV exposure survival analysis revealed similar results for all the different strains and a higher surviving fraction in the case of wild-type *E. coli* when compared to *D. radiodurans*. This UV fluence exposure reached ~ 30% of the UV fluence observed on the Exposure Facility of the ISS per minute, so further exposures to higher doses could be done in order to analyze the limitations of the new strains.

A further prolongation of the directed evolution process by adding additional cycles and even exposing the cells to higher doses of ionizing radiation or changing the dose rate could lead to an improvement of the surviving fraction of the *E. coli* Dsup strain. In addition, X-rays were chosen due to their high energy and penetration capacities, but exposing the cells to ionizing radiation types such as gamma or neutron radiation could be important to test the survival of the cells under LEO environmental conditions. Higher UV doses could also be tested to further examine the limits of the strains developed during the study.

The vacuum and pressure tests revealed the insignificance of cell aggregation for *E. coli*'s survival. While *D. radiodurans* showed an increase in the surviving fraction after being aggregated of approximately 3 orders of magnitude with respect to the non-aggregated sample, *E. coli*'s survival showed no significant variation with or without aggregation after spending 3 days at 7 kPa and being exposed to three temperature cycles. These results are consistent with the rest of the temperature and pressure conditions tested for *E. coli* and *D.radiodurans* wild-types.

With respect to the effects of the cells transformed with the genes encoding for RecA, uvrD, and Dsup, the surviving fraction decreased for all the selected colonies of the different *E. coli* transformed strains in comparison with the wild-type *E. coli*, with similar values and responses. The exposure of the cells to 3 days under 7 kPa showed the best survival results, with lower surviving fractions for cells exposed to the temperature cycles after 0 or 7 days under 7 kPa of pressure. These results could be due to the activation of the DNA repair mechanisms during the vacuum exposure and being already active during the beginning of the temperature cycles. However, after a longer period of vacuum exposure, the DNA repair mechanisms start to lose their effectiveness.

To further study the limitations of the studied strains, as well as other bacteria, directed evolution processes could be applied to enhance the survival of the cells to vacuum or high/low temperatures. Moreover, some genes from extremophile species have been identified and related to thermal resistance (Gonzalez-Blasco et al., 2000; Zhang and Griffiths, 2003; Ritter et al., 2014), which could be introduced to the cells and characterized. However, thermal and vacuum resistance is not only a matter of genetics, but also of cell components and protein melting and degradation. Therefore, selecting a bacteria already capable of surviving in similar conditions could be the best option to develop extra-terrestrial habitats.

The studied salinity values showed no major impairments for the growth of the tested *E. coli* strains, with similar growths in all the cases from 0 to 20 g/l of sodium chloride. In the case of pH, the viability range for all the strains showed to be between 5 and 9, with no growth for either pH 3 or 11. These results are consistent with previous studies on *E. coli*'s growth in media with different pH concentrations (Ross et al., 2003). The *E. coli* RecA strain needed more time to reach the mid-exponential phase at pH 5. However, this difference is not considered significant.

This study has evidenced that an enhancement of cellular resistance to harsh environments is possible but is also limited by the current knowledge on the natural genetic and molecular mechanisms of protection and DNA repair existing in extremophiles. Consequently, increasing the knowledge on these mechanisms is still key to develop resistant bacterial strains able to survive under extreme conditions and develop extra-terrestrial habitats. In this study, the cellular resistance of *E. coli* was enhanced to the point that it should allow the survival of some of the selected strains in environments like the equator of Mars, but also outside of the International Space Station in the Low Earth Orbit. New tests and procedures should be performed to further study and characterize the surviving possibilities and limitations of the evolved strains. For instance, the biological shielding properties of cell aggregation could increase the survival to UV radiation, but also to corpuscular Ionizing Radiation, such as beta or gamma rays. Shielding bacteria from radiation, especially UV, has proven to be very effective for the survival of *B. subtilis* spores in space (Horneck et al., 1994). As for charged particles radiation, shielding could also be beneficial, as proved in previous studies with *D. radiodurans* (Paulino-Lima et al., 2011). Also, a combination between vacuum and continuous wide temperature cycles exposure should be done to further characterize and potentially expand the limits of the selected strains.

The development of environments hospitable to life and the possibilities of building extra-terrestrial habitats are tied to the knowledge and capacity of enhancing bacterial, fungi, and cellular resistance to the existent conditions. For this, many environmental factors must be considered, but also some ethical and moral concerns. The selection of the most adequate species for the development of the desired ecosystem has a major role, and the capacity of controlling the survival of the introduced species is key for the preservation of the natural conditions. The preservation of environments and the avoidance of microbe transfer has led to many serviceable spacecrafts and satellites being terminated to minimize the risks of microbe transfer, as was the case of NASA's orbiter Cassini (Yam et al., 2009), which was burned in Saturn's atmosphere before an eventual crash into Enceladus, which has been studied as an eminently habitable environment. These procedures comply with the planetary protection protocols, established to minimize the risks of depositing Earth microbes into possibly habitable environments. However, it has been proven impossible to construct a microbe-free spacecraft, and experiments such as the ones done with *D. radiodurans* prove that some species can remain viable in outer space. These studies have led to very strict policies on space exploration, but still, the risks are not eliminated. In addition, the idea of human exploration adds more concerns to this field, and any human mission in space would almost certainly have to break those policies to survive. On the other hand, space exploration is not the only source of microbe transfer, as debris caused by asteroid impacts could also carry potentially viable microbes. All this leads to the conclusion that eventual microbe transfer is inevitable unless space exploration is abandoned, and no exchange between other planets or organic matter is happening.

Space exploration is essential for survival, expanding not only our knowledge and resources, but also opening the door to many new possibilities. However, as this study has revealed, there is still plenty of knowledge and resources to be gained on Earth that could help in the exploration and development of extra-terrestrial habitats. Additionally, many life science engineering strategies could be potentially used in this field, and the search of Earth's life limits and their expansion could provide some insight into the generation of habitable environments. Space is believed to be the last frontier, but the truth is, we are still a frontier to ourselves.

## Data Availability Statement

The datasets presented in this study can be found in online repositories. The names of the repository/repositories and accession number(s) can be found at: https://www.ebi.ac.uk/ena, PRJEB47882.

## Author Contributions

JP performed the experiments with help of NK and supervision of MG. The study was designed by JP, NK, and MG. MA and JQ helped and supervised the ionizing radiation exposures. JP, NK, JQ, MA, and MG wrote the manuscript. All authors contributed to the article and approved the submitted version.

## Funding

This study has received funds from Captació Talent - Fundació La Caixa, the Office of Naval Research (Award N62909-18-1-2155), Ramón y Cajal program (Grant agreement RYC-2015-17734). NK is funded by the Sociedad Española de Quimicos Cosmeticos (SEQC) and thanks the SEQC for the participation at the 33rd IFSCC Congress.

## Conflict of Interest

The authors declare that the research was conducted in the absence of any commercial or financial relationships that could be construed as a potential conflict of interest.

## Publisher's Note

All claims expressed in this article are solely those of the authors and do not necessarily represent those of their affiliated organizations, or those of the publisher, the editors and the reviewers. Any product that may be evaluated in this article, or claim that may be made by its manufacturer, is not guaranteed or endorsed by the publisher.
