# Supplementary material for: DNA Damage Protection for Enhanced Bacterial Survival Under Simulated Low Earth Orbit Environmental Conditions in Escherichia coli
Source: Front Microbiol. 2021 Dec 14;12:789668. doi: 10.3389/fmicb.2021.789668 (PMC8713957; doi:10.3389/fmicb.2021.789668)
Supplement: Supplementary file 1 [file Data_Sheet_1.PDF]

frontiers

Supplementary Material

1 DNA PRIMER TABLES

Table S1. Primers used in the amplification reactions of the pBAD, RecA, uvrD, and Dsup genes using *KAPA HiFi HotStart ReadyMix* (Roche) and the addition of the desired restrictions sites for the subsequent cloning.

Gene	Primer name	Sequence (5' - 3')
**pBAD/AraC**	pAra_EcorI_fwd<br>pAra_XbaI_rev	*tttctggaattcttatgacaacttgacggctacatca*<br>*catacccgtttttttgggctagctctagaatacgta*
**RecA**	RecA_SpeI_fwd<br>RecA_PstI_rev	*agcatcactagtatgagcaaggacgccaccaaa*<br>*cgaagccgccgaagcgtaactgcagtccggcaaa*
**uvrD**	uvrD_SpeI_fwd<br>uvrD_PstI_rev	*atcgtctactagtatgacttcttccgccggcc*<br>*ccaacctgacgaagctgtagctgcagtccggca*
**Dsup**	Dsup_XbaI_fwd<br>Dsup_SpeI_rev	*gccgcttctagaatggcatccacacaccaatcatc*<br>*tggaggacggaagaggaagtaaactagtagctta*

Table S2. Primers used in the protein expression analysis of the RecA, uvrD, and Dsup genes by means of qPCR.

Gene	Primer name	Sequence (5' - 3')
**RecA (*E. coli*)**	EcRecA_qPCR2_fwd<br>EcRecA_qPCR2_rev	*gatatcgacaacctgctgtgc*<br>*agcagcgtgttggactgctt*
**RecA (*D. radiodurans*)**	DrRecA_qPCR1_fwd<br>DrRecA_qPCR1_rev	*tcgaaacagccatgagccag*<br>*accagcagttcgtcggtgtt*
**uvrD (*D. radiodurans*)**	DruvrD_qPCR3_fwd<br>DruvrD_qPCR3_rev	*aggaggagcggcgactctt*<br>*gtgttcttgaccgctgtcgt*
**Dsup (*R. varieornatus*)**	RvDsup_qPCR1_fwd<br>RvDsup_qPCR1_rev	*cagaaccctcttccacaggt*<br>*gggacttctttggctgggc*

2 QPCR RESULTS

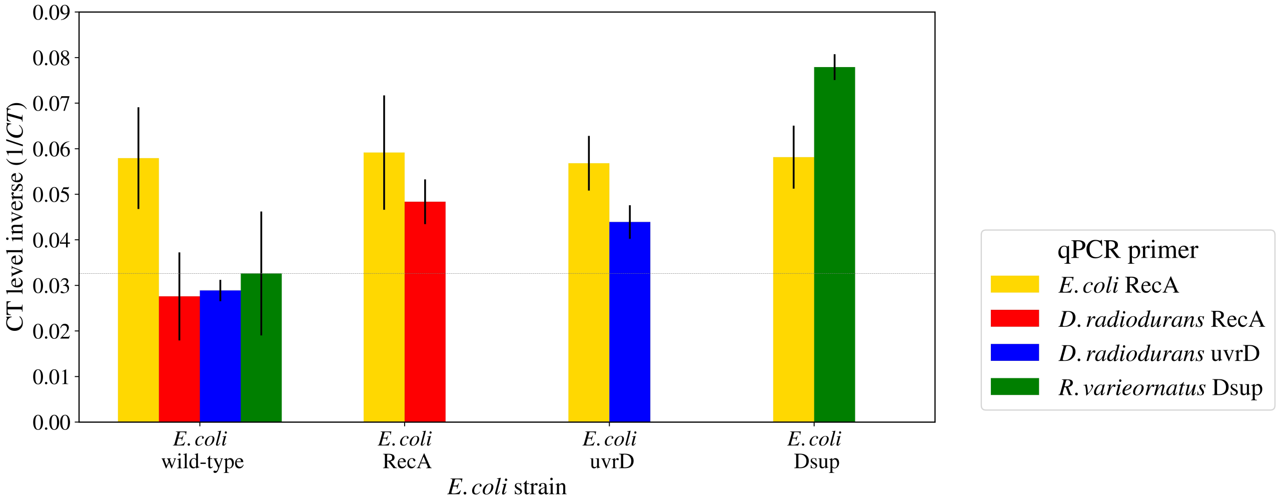

Figure S1. CT values for the *D. radiodurans* RecA and uvrD, and *R. varieornatus* Dsup genes in each strain. *E. coli*'s RecA gene was used as a housekeeping gene of reference for gene transcription.

3 WHOLE GENOME SEQUENCING: PREDICTED MUTATIONS ANALYSIS

Table S3. Whole Genome Sequencing predicted mutations compared with the wild-type *E. coli* K-12 MG1655. The analysis was done using the breseq software from Barrick Lab (The University of Texas at Austin, 2014). For the three inserted genes, one colony from RecA, one from uvrD and three from Dsup were selected for this sequence analysis. One of the Dsup colonies (C15) showed no predicted mutations.

**Predicted mutations**			
***E. coli* strain**	**Position**	**Mutation**	**Gene**
***E. coli* RecA**	131,040	C → T	yacH ←
	556,950	G → A	folD ←
	1,495,856	G → A	ydcJ →
	2,012,180	T → A	yedL → / ← fliE
	3,043,720	C → T	bglA →
***E. coli* uvrD**	1,196,220	C → T	icd →
	1,196,232	C → T	icd →
	1,196,245	T → C	icd →
	1,196,247	A → G	icd →
	1,365,179	G → A	puuB →
	1,372,385	Δ1 bp	ycjN →
	1,372,386	G → T	ycjN →
	1,574,415	T → A	pqqL ←
	1,638,801	G → T	cspI ← / ← rzpQ
	2,186,179	C → A	rcnA →
	3,661,946	C → T	mdtF →
	4,475,002	T → C	tabA →
***E. coli* Dsup C16**	444,473	A → G	panE ←
	480,073	C → A	maa ← / ← hha
	550,379	G → T	ylbF →
	2,386,067	A → G	yfbL →
***E. coli* Dsup C17**	1,710,983	C → T	rsxE →
	2,230,076	T → C	dusC ←
	4,020,980	C → T	ubiB →
